# Changes in children’s wellbeing in Bradford during COVID-19: The Born in Bradford COVID-19 longitudinal research study

**DOI:** 10.12688/wellcomeopenres.17642.2

**Published:** 2022-05-30

**Authors:** Katie Pybus, Brian Kelly, Bo Hou, Mildred Ajebon, Claire McIvor, Daniel Bingham, Rosemary McEachan, Kate E. Pickett, Josie Dickerson

**Affiliations:** 1 Department of Health Sciences, University of York, UK, York, UK; 2 Bradford Institute for Health Research, Bradford Hospitals National Health Service Trust, Bradford, UK

**Keywords:** children, wellbeing, mental health, COVID-19, pandemic

## Abstract

**Background: **Concerns have been raised about the potential impact of COVID-19 and associated lockdown measures on child mental wellbeing, but emerging evidence suggests mixed results and there is a dearth of information from ethnically diverse samples. The current study aims to explore the impact of the pandemic on wellbeing using longitudinal data collected from the multi-ethnic Born in Bradford family cohort study.

**Methods: **Within-child changes in wellbeing were explored using data collected pre-pandemic and again during the first UK lockdown for 500 children aged 7-13 from a range of ethnic and socioeconomic backgrounds, using self-reported feelings of happiness and sadness. Associations between changes in wellbeing, demographic factors, quality of social relationships and physical activity levels were explored using multinomial logistic regression models.

**Results: **In this sample, 55% of children reported no change in their wellbeing from pre-pandemic to during the first lockdown (n=264). Children of Pakistani heritage were more than twice as likely to report feeling sad less often than White British children (RRR: 2.61, 95% CI: 1.23, 5.51) during the first lockdown. Those who reported being left out by other children before the pandemic were over three times as likely than those who did not (RRR: 3.72: 1.51, 9.20) to report feeling sad less often during the pandemic. Around a third of children reported feeling happier (n=152, 31.6%), but these changes did not relate to any of the explanatory variables included in this analysis.

**Conclusion:** Many children in this study reported no changes in their wellbeing during the first UK lockdown compared to before the pandemic and some described improved wellbeing. These findings suggest that children have coped well with the significant changes over the past year, though targeted support, particularly for those children who felt excluded before the pandemic, would be beneficial.

## Background

Prior to the COVID-19 pandemic, rates of mental illness amongst children and adolescents in the United Kingdom (UK) appeared to be rising. Evidence suggests that in 2017, 12.5% of children and young people aged between 5 and 19 met the criteria for at least one mental health problem, and that the prevalence of mental ill health has been increasing over time ( Public Health England, 2019). Experiencing a mental health problem in childhood can impact on multiple areas of development, and have longer-term health and social implications, meaning that early intervention and prevention is key ( Mental Health Foundation, 2019).

In March 2020, the UK entered the first of multiple national lockdowns to curtail the spread of COVID-19. This included having to work from home (or being unable to work, as non-essential businesses were closed during this time), the closure of schools and non-essential shops, as well as being able to leave the house only for daily exercise, essential shopping, or for emergencies. Pre-existing health inequalities were swiftly ‘exposed and exacerbated’ by the emergence of COVID-19, with those living in socioeconomically deprived circumstances, as well as people from ethnic minority backgrounds, more at risk of being hospitalised and of dying from the virus ( Marmot *et al.*, 2020). In addition, the impact of national lockdowns on financial insecurity and mental health was unequal, with the largest effects among those already vulnerable, including ethnic minorities and those living in socioeconomically deprived areas ( Dickerson *et al.*, 2020; Marmot *et al.*, 2020; NatCen, 2021).

For families with children, the closure of educational settings further impacted on daily life. Parents and schools had to adapt quickly to online modes of learning and home-schooling, a challenge that brought with it additional costs for families and increased pressure on the already stretched budgets of low-income households ( Brewer & Patrick, 2021) as well as stress and tension in the home ( Creswell *et al.*, 2021; Dickerson *et al.*, 2020). Adult mental health deteriorated during the first lockdown compared to pre-pandemic levels, particularly amongst younger adults, women, those with young children in the household and those who were financially insecure ( Dickerson *et al.*, 2021; Pierce *et al.*, 2020).

Meanwhile for children and young people, concerns have been raised about the potential for longer-term educational inequalities to emerge due to uneven schooling experiences during the pandemic ( Mon-Williams *et al.*, 2021). In addition, there have been concerns about the impact of restrictions and a lack of social contact on child mental health ( Rajmil *et al.*, 2021). Emerging evidence suggests a high prevalence of COVID-19-related fears amongst children and adolescents ( Samji *et al.*, 2021). At the same time, access to protective factors that can help to improve wellbeing such as physical activity and access to green space were affected by COVID-19 restrictions ( Bingham *et al.*, 2021; Masterton *et al.*, 2020).

Yet despite predictions about the negative impact of the pandemic on child mental wellbeing, evidence so far suggests mixed effects, with some research finding a deterioration in wellbeing, whilst other studies have found evidence of improved wellbeing or no overall change - though it has been difficult as yet to obtain definitive longitudinal data including pre-pandemic measures, and data that is representative of ethnic and socioeconomic diversity ( Ford *et al.*, 2021; Lewis & Bunn, 2021; Office for Health Improvement and Disparities, 2021). Parent-reported measures (Strengths and Difficulties Questionnaire) elicited at two timepoints during the first lockdown did suggest deteriorating mental health amongst children aged 4–10 in the UK, as evidenced by a 10% increase in emotional symptoms, a 20% increase in hyperactivity/inattention and a 35% increase in conduct problems, but this sample is disproportionately White British and higher income ( Shum *et al.*, 2021; Waite *et al.*, 2021), and parent perspectives may differ from those of children themselves.

Initial follow up survey data from the longitudinal Mental Health of Children and Young People in England survey undertaken in July 2020 also found an increase in the incidence of probable mental health problems from 11% to 16% across children aged 5–16 ( Newlove-Delgado *et al.*, 2021), though little is currently known about differences in wellbeing between children during the pandemic and the factors influencing this. Bignardi *et al.* (2021) found increases in depression amongst children aged 8–12 during the first lockdown compared to pre-pandemic, but this study did not report on differences by ethnic group. The experiences of ethnic minority children remain under-represented in research focusing on child and adolescent mental health during the pandemic more broadly ( Lewis & Bunn, 2021), part of a wider issue relating to underrepresentation of ethnic minority participants in COVID-19 research ( Etti *et al.*, 2021). In the context of pre-existing health inequalities affecting people from ethnic minority backgrounds, which have been made worse by the pandemic, it is especially important to understand how wellbeing differs for children by ethnicity, to ensure appropriate interventions can be put in place to reduce inequalities.

Throughout the pandemic and as part of the ongoing Born in Bradford COVID-19 longitudinal research programme, a series of surveys were undertaken with children in Bradford, a city in the North of England ( McEachan *et al.*, 2020). Bradford is the fifth largest metropolitan district in England and contains an ethnically diverse population with high levels of socioeconomic deprivation. In the 2011 census, 64% of the population were of a White British background and the second largest ethnic group (20%) is people of Pakistani heritage ( City of Bradford Council, 2018). Within the Born in Bradford study, and just prior to the pandemic, some children had participated in studies collecting socio-demographic, socio-economic and wellbeing data. This particular data is able to speak to broader changes in mental wellbeing across this age group and in our study, we do not seek to determine clinically significant levels of mental health difficulties. The data offered an opportunity to describe how child wellbeing changed from before the pandemic to during the first UK national lockdown, as well as to gain an understanding of the drivers of these changes in an ethnically diverse population. Keeping in mind concerns about the lack of social contact on child wellbeing during the pandemic, see for example, ( Rajmil *et al.*, 2021), we sought to assess whether the quality of social relationships, that is how well the child describes getting on with family and peers, had any relationship with wellbeing.

### Aims

To understand whether self-reported wellbeing has changed amongst children in Bradford from pre-pandemic to during the first UK national lockdown.

To understand what demographic factors are associated with these changes in relation to age, gender, ethnicity and socioeconomic deprivation.

To understand whether the quality of social relationships in the pre-pandemic time period is associated with changes in wellbeing during the pandemic amongst children.

## Methods

As part of the Bradford COVID-19 response, children of primary and early secondary school age (7–13) already participating in the Born in Bradford Growing up study (see Bird *et al.*, 2019 for further details) were surveyed by post during the first national lockdown (May to July 2020). The survey included measures of wellbeing for which data were also available for 500 children from before the pandemic, alongside information on physical activity levels and quality of social relationships – factors with the potential to impact on wellbeing outcomes.

### Setting

Born in Bradford (BiB) is a family cohort study which recruited 12,453 pregnant women with 13,376 pregnancies between March 2007 and November 2010 and has subsequently studied both short and long-term health and social outcomes across multiple domains in this population ( Wright *et al.*, 2013). Two recent studies had collected data on the BiB children and their families prior to the pandemic: BiB Growing Up (BiBGU), which collected data from BiB cohort families in community-based assessments between 2017–2020, and BiB Primary School Years (PSY), which collected data from children aged 6–11 in school-based assessments between 2016–2019 (see Bird *et al.*, 2019). At the time that the BiB COVID-19 study took place, 5,300 children had participated in BiBGU and, 15,641 children from 89 primary schools participated in PSY, of whom 6,147 were BiB children ( Pickett *et al.*, 2021).

Social and emotional wellbeing was assessed within the PSY study. Parents and carers of eligible children were sent information sheets by post and consent was obtained on an ‘opt-out’ basis, with the survey itself being completed in educational settings. Ethical approval was obtained from the National Health Service Health Research Authority Yorkshire and the Humber (Bradford Leeds) Research Ethics Committee (reference: 16/YH/0062). Children self-completed a survey (“Me and My Life”), developed using questions derived from the Millennium Cohort Study, the International Survey of Children’s Wellbeing and the Avon Longitudinal Study of Parents and Children ( Pickett *et al.*, 2021). Amongst these questions, children provided information on self-reported wellbeing by being asked how often they felt happy and sad with potential response categories: always, sometimes, never. A copy of the survey is available as extended data associated with this article. 

In May 2020, during the first UK national lockdown, 5,300 COVID-19 questionnaires were sent out by post, alongside information sheets for both caregivers and children, to children who had participated in BiBGU. A copy of the survey is available as extended data associated with this article. The first UK lockdown began on 20 ^th^ March, 2020 and so most children (excluding children of key workers and children considered vulnerable) had spent the majority of their time at home, and were unable to attend their usual school environment or interact with peers face to face for two to three months at the time of completing the questionnaire.

The overall response rate for this COVID Phase 1 child survey was 18.3% (N=970). This study was approved by the National Health Service Health Research Authority Yorkshire and the Humber (Bradford Leeds) Research Ethics Committee (Substantial amendments to: BiBGU 16/YH/0320 and BiBBS 15/YH/0455). For this survey, and as approved by the Health Research Authority and Bradford/Leeds research ethics committee, implied consent was assumed for all questionnaires completed via post or online. Children were asked about a range of different topic areas including physical activity, sleep and sedentary behaviour, education and home-schooling, social relationships, wellbeing and food security. The same questions regarding self-reported happiness and sadness as used in the PSY study were used to explore child wellbeing in the COVID-19 survey, enabling exploration of wellbeing pre-and during the pandemic for those children who took part in both studies. Information on children who participated in both PSY and the COVID Phase 1 Survey was linked using unique identifiers for the purposes of this research.

### Participants

Children who participated in the first COVID-19 survey and had linked data available from the Primary School Years study were included in this analysis (n=500).

### Study measures

Self-reported wellbeing was measured using the responses to two questions: How often do you feel sad?’ and ‘How often do you feel happy?’ with three answer options of ‘always’, ‘sometimes’ or ‘never’. These questions were derived from the Millennium Cohort Study self-completion child questionnaire which underwent piloting prior to main data collection ( Centre for Longitudinal Studies, 2020). In addition, think aloud testing with children of different ages and ethnicities was carried out by the Born in Bradford research team during the development of the ‘Me and My Life’ survey, adapting any questions that children found difficult to understand as required ( Bird *et al.*, 2019).

To measure change in subjective wellbeing between the two timepoints a new variable was derived from the above categorical response data using conditional expressions to generate three outcome categories: no change in self-reported feelings of sadness, negative change (reported feeling sad more often) and positive change (reported feeling sad less often). For example, children who reported pre-pandemic that they never felt sad but who subsequently reported in the first lockdown that they sometimes or always felt sad would be classified as experiencing a negative change. The same method was applied to changes in self-reported feelings of happiness: no change in self-reported feelings of happiness, negative change (reported feeling happy less often) and positive change (reported feeling happy more often) between the two timepoints.

Gender, age, ethnicity and socioeconomic deprivation are all known to be associated with mental wellbeing ( Mental Health Foundation, 2022) and so were included as demographic factors. Physical activity levels during the pandemic were included as a potential explanatory variable in keeping with findings relating to outdoor activity, wellbeing and the pandemic ( Bingham *et al.*, 2021). Finally, the quality of social relationships from before the pandemic was included as a variable which might potentially help explain positive and negative changes in subjective wellbeing under COVID-19 restrictions on social contact ( Rajmil *et al.*, 2021). All pre-pandemic measures were collected between 2016 and 2019.

Socioeconomic deprivation was measured by deriving quintiles of the 2019 Index of Multiple Deprivation ( Ministry for Housing, Communities and Local Government, 2019). The Index of Multiple Deprivation (IMD) represents neighbourhood, rather than individual, level of socioeconomic deprivation, however this was the most comparable measure available across the datasets. Ethnicity was recoded from the 2011 Census categories into a three-category variable representing the two largest ethnic groups in the city: Pakistani heritage and White British, as well as an ‘Other’ category to capture children from a wide range of additional ethnic groups present in the sample. 

Physical activity levels were measured by a modified version of the validated seven day recall questionnaire, the Youth Activity Profile-English Youth Version (YAP) ( Fairclough *et al.*, 2019; Saint-Maurice & Welk, 2015). The variable ‘meeting physical activity guidelines’ was derived on whether children reported they had taken part in 60 minutes or more of moderate-to-vigorous physical activity on a usual weekday and weekend day in the previous week. 

Quality of social relationships was measured via questions asking about relationships with family members and with peers. Family relationships were measured using self-reported perception of how well the family gets along; while quality of peer relationships was explored using two variables: how often the child felt left out by other children and experiences of bullying.

### Data analysis

Demographic data were first explored using descriptive statistics to understand the sample characteristics and to describe wellbeing for the study population pre-pandemic to during the first lockdown.

Within-child changes in self-reported wellbeing were explored using cross-tabulation to compare survey responses pre-pandemic and during the first lockdown (May to June 2020).

Multinomial logistic regression models were estimated for change in subjective wellbeing, first including only demographic factors as explanatory variables, with additional explanatory variables then being added. Relative risk ratios and marginal effects were estimated ( Mize, 2019).

All statistical analysis was undertaken using Stata 16.1 ( Statacorp, 2019).

## Results

Demographic characteristics of the sample are reported in Table 1, alongside a comparison with BiB children who were part of the PSY sample. There were no missing data for gender, IMD or age in the study sample but a small number of children (n=12, 2.4%) did not have data recorded for ethnicity. Compared to the PSY (pre-pandemic) survey, there is an under-representation of children of Pakistani heritage in terms of those who completed both surveys – 63.6% of children were from this ethnic background in the original PSY sample, compared to 56.1% of children completing surveys both pre- and during the pandemic, and an over-representation of those from ‘Other’ ethnic backgrounds – 15.0% of those who completed both surveys, compared to 7.2% of BiB children in the PSY study. The White British sample, as well as the gender distribution remains broadly the same. Data on Index of Multiple Deprivation suggest that fewer of those completing both surveys lived in neighbourhoods in the most deprived quintile – 46.0% of respondents in the sample for this study, compared with 66.5% of BiB children for whom IMD is available (n=5,138) in the original PSY sample.

**Table 1.** Sample characteristics.

	Study sample N (%)	Primary School Years N (%)
**Gender**		
Male	248 (49.6%)	3140 (51.1%)
Female	252 (50.4%)	3007 (48.9%)
Missing	0	0
Total	500	6147
**Ethnicity**		
White British	141 (28.9%)	1695 (29.1%)
Pakistani heritage	274 (56.1%)	3695 (63.6%)
Other	73 (15.0%)	421 (7.2%)
Missing/Unknown	12	336
Total	500	6147
**Age**		
**Age (mean, SD):** pre- pandemic	7.9 (7.2 – 8.7)	7.9 (7.2 – 8.6)
**Age (mean, SD):** during pandemic	10.2 (9.2 – 11.1)	Not applicable
Missing	0	0
Total	500	6147
Index of Multiple Deprivation*		
1 (most deprived)	230 (46.0%)	3,535 (66.5%)
2	84 (16.8%)	934 (17.6%)
3	90 (18.0%)	583 (11.0%)
4	47 (9.4%)	167 (3.1%)
5 (least deprived)	49 (9.8%)	99 (1.9%)
Missing	0	829
Total	500	6,147

T-test for difference in means: age p= 1.000. Chi square tests for categorical variable differences: gender p = 0.524, ethnicity p < 0.001, IMD p < 0.001

### Study population - subjective wellbeing

Self-reported experiences of happiness and sadness at each time point are presented in Table 2 and Table 3. Some respondents did not record a response for individual questions and so were excluded from each relevant analysis (see valid N), this totalled no more than 14 children in any single instance.

**Table 2.** Self-reported feelings of happiness pre-pandemic and during the pandemic.

How often do you feel happy?			
	Pre-pandemic Valid N=494	During the pandemic Valid N=486	Population Change
All of the time	179 (36.2%)	259 (53.2%)	+17%
Sometimes	302 (61.1%)	221 (45.5%)	-16.3%
Never	13 (2.6%)	6 (1.2%)	-1.2%
Missing	6	14	
Total	500 (100%)	500 (100%)	

**Table 3.** Self-reported sadness pre-pandemic and during the pandemic.

How often do you feel sad?			
	Pre-pandemic Valid N=490	During the pandemic Valid N=487	Population Change
All of the time	20 (4.1%)	7 (1.4%)	-2.6%
Sometimes	370 (75.5%)	357 (73.3%)	-2.2%
Never	100 (20.4%)	123 (25.3%)	+ 4.8%
Missing	10	13	
Total	500 (100%)	500 (100%)	

There was not much change in self-reported feelings of sadness across the two time points: there was a small increase of 4.8% of children reporting that they never feel sad during the lockdown. The proportion of change in feelings of happiness were much larger: the proportion of children who reported feeling happy all of the time rose from 36.2% to 53.2% during the lockdown.

### Within-child changes in subjective wellbeing

Cross-tabulation was used to understand changes in subjective wellbeing (self-reported happiness and sadness) pre-pandemic compared to during the first UK lockdown, see extended data. In relation to feeling both happy and sad, by far the largest number of children reported no changes: 67.1% (n=320) reported no changes in feelings of sadness and 55.0% (n=264) of children reported no changes in their feelings of happiness.

13.6% (n=65) of children reported feeling sad more often during the first lockdown compared to pre-pandemic and 19.3% (n=92) reported feeling sad less often.

31.7% of children reported feeling happy more often during the pandemic than pre-pandemic (n=152), whilst a small number 13.3% (n=64) reported feeling happy less often during the pandemic.

### Multinomial logistic regression models

**Figure 1.**
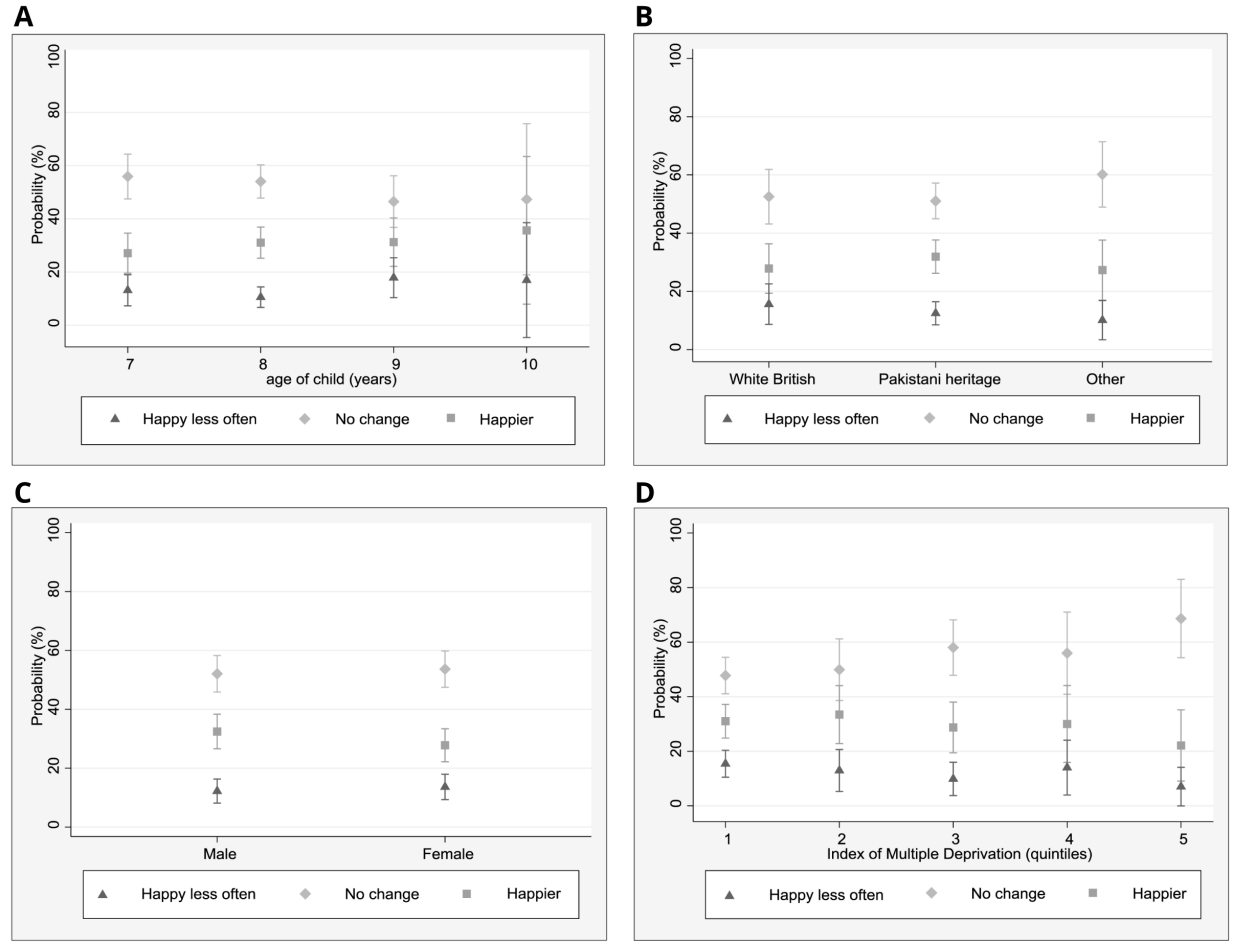
Marginal effects plots: happiness. **A**. Predicted margins for age and happiness (95% CI) **B**. Predicted margins for ethnicity and happiness (95% CI) **C**. Predicted margins for gender and happiness (95% CI) **D**. Predicted margins for IMD quintiles and happiness (95%(CI).

**Figure 2.**
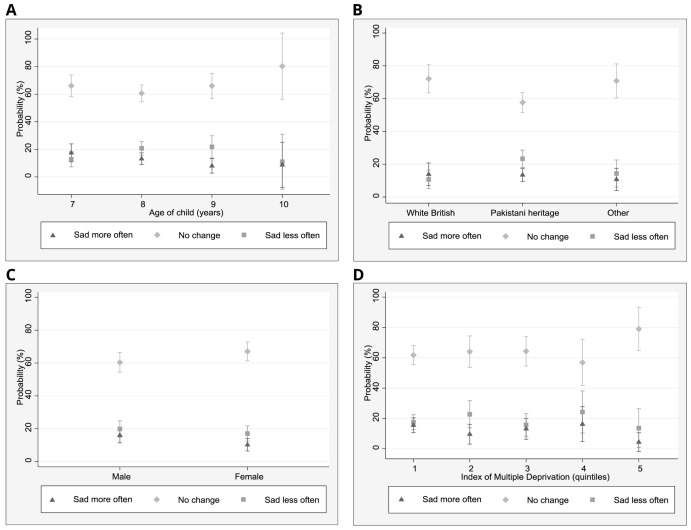
Marginal effects plots: sadness. **A**. Predicted margins for age and sadness (95% CI) **B**. Predicted margins for ethnicity and sadness (95% CI) **C**. Predicted margins for gender and sadness (95% CI) **D**. Predicted margins for IMD quintile and sadness (95% CI).

To better understand the role of demographic factors in relation to self-reported changes in sadness and happiness, marginal effects were estimated for each of the multinomial logistic regression models to assist with the interpretation of model results using effect sizes rather than coefficients ( Figure 1 and Figure 2). Demographic measures are pre-pandemic. In relation to the change ‘feeling sad less often’, 23% of children of Pakistani heritage (95% CI: 18–29%) reported this, whilst 11% (5–27%) of White British children and 14% (6–23%) of children from other ethnic groups described feeling sad less often. No differences in changes in sadness were found in relation to age and IMD.

Multinomial logistic regression models were subsequently expanded to explore the relationship between demographic characteristics, quality of social relationships and changes to sadness and happiness over time. All explanatory variables, excluding change in wellbeing over time and physical activity levels, are pre-pandemic measures.

### Self-reported feelings of happiness

On inspection of the model results, no statistically significant (at 95% CI) associations were observed between changes in self-reported happiness and any of the explanatory variables. These findings are therefore not discussed in detail here, however, the related multinomial models are available as extended data to this article as well as models of fit for self-reported sadness.

### Self-reported feelings of sadness

Table 4 provides a summary of findings for changes in self-reported sadness comparing each outcome (sad more/less often), full models are available as extended data. There was no association (95% CI) in this sample between feeling sad less often over time with gender or with socioeconomic status. Children of Pakistani heritage were, however, more than twice as likely to report feeling sad less often during the first lockdown than White British children (RRR: 2.61, 95% CI: 1.23, 5.51). Children who met weekly physical activity guidelines were more likely to report changes to their reported feelings of sadness during the lockdown, compared to those who did not, but this association was not statistically significant (sad less often: 0.64: 0.35, 1.17; sad more often: 0.59, 0.30, 1.18).

**Table 4.** Self-reported changes in feelings of sadness compared to no change (base) between pre-pandemic and during the pandemic (RRR).

	Sad less often	Sad more often
**Gender**		
Female (base)	-	-
Male	1.25 (0.74–2.12)	1.45 (0.78–2.69)
**Ethnicity**		
White British (base)	-	-
Pakistani	2.61 (1.23–5.51) *	1.10 (0.50–2.44)
Other	1.27 (0.47–3.38)	0.76 (0.28–2.11)
**Age**		
7 (base)	-	-
8	2.20 (1.13-4.26) **	0.90 (0.46–1.73)
9	1.99 (0.92–4.29)	0.46 (0.18–1.15)
10	0.72 (0.07–6.78)	0.31 (0.03–2.83)
**IMD quintile**		
1 (base)	-	-
2	1.38 (0.70–2.73)	0.56 (0.22–1.41)
3	0.97 (0.47–2.00)	0.82 (0.37–1.79)
4	1.78 (0.69–4.60)	1.35 (0.48–3.75)
5	0.65 (0.19–2.23)	0.25 (0.05–1.25)
**Meet physical activity guidelines?**		
No (base)	-	-
Yes	0.64 (0.35–1.17)	0.59 (0.30–1.18)
**How often does your family get along well together?**		
Never (base)	-	-
Some of the time	0.43 (0.11–1.71)	0.43 (0.07–2.43)
All of the time	0.41 (0.10–1.66)	0.74 (0.13–4.13)
**How often are you left out by other children?**		
Never (base)	-	-
Some of the time	1.03 (0.56–1.91)	0.44 (0.22–0.86) *
All of the time	3.72 (1.51–9.20) **	0.96 (0.30–3.09)
**How often are you bullied by other children?**		
Never (base)	-	-
Some of the time	0.59 (0.33–1.04)	1.00 (0.53–1.89)
All of the time	0.77 (0.29–1.99)	0.84 (0.22–3.12)

*P < 0.05, ** *P* < 0.01, *** *P* < 0.001. Note: All variables are pre-pandemic measures, aside from physical activity levels.

Children who, before the pandemic, reported feeling left out by other children all of the time, as compared to being left of out some of the time or not at all, also reported feeling sad less often during the pandemic (3.72: 1.51, 9.20). Other measures of social relationships – ‘how often does your family get along well together?’ and ‘how often are you bullied by other children?’ – did not, however, have an independent association with feeling sad less often during the pandemic compared to pre-pandemic, but their inclusion in the model did appear to strengthen the association with both ethnicity and age (see extended data).

Table 3 additionally reports on the likelihood of children feeling sad more often. Male children were more likely to report feeling sad more often than females during the pandemic compared to pre-pandemic (1.75: 1.01, 3.07 – extended data) but this association became non-significant (95% CI) once social relationships were included in the model. In the final model which includes all social relationship variables, this association becomes weaker but here, children who reported being left out by other children some of the time, compared to not at all, before the pandemic were more likely to report having experienced no change in their feelings of sadness compared to feeling sad more often (0.44: 0.22, 0.86) during the pandemic.

## Discussion

### Key findings

Overall, we found that children aged 7–13 living in Bradford reported improved levels of happiness during the first UK lockdown compared to pre-pandemic. In this sample, there appeared to be little effect of demographic and social relationship factors on changes in self-reported happiness.

In contrast, whilst changes in levels of sadness were smaller, there were some differences across demographic and social relationship factors. Children of Pakistani heritage were more likely to report improved wellbeing (feeling sad less often) during the pandemic compared to White British children, and males had a greater likelihood of reduced wellbeing (feeling sad more often) compared to females, though this difference became non-significant once social relationship variables were added to the model. Social relationships – particularly feeling left out by other children – appear to account for part of the relationship between demographic factors and changes in feelings of sadness. Children who reported feeling left out by other children all of the time before the pandemic reported feeling sad less often during the pandemic.

Research on the impact of COVID-19 on child mental wellbeing continues to emerge and is in the early stages at present given the comparatively short time frame since the pandemic took hold in earnest. These findings do, however, mirror those of other studies that have found fewer than expected changes to child wellbeing during the pandemic though the evidence so far remains mixed overall ( Ford *et al.*, 2021; ImpactEd, 2021). Research with slightly older children – those aged 13–14, found decreased anxiety, improved wellbeing and no large change in risk of depression in the first lockdown compared to pre-pandemic, and those with lower social connectedness to school, family and friends before the pandemic were more likely to report improved wellbeing during the first lockdown, compared to those with higher connectedness ( Widnell *et al.*, 2020).

### Implications of findings

It is of some consolation to note that in a period that saw significant and often life changing disruptions to daily life, the majority of children – in this sample at least – appeared to be relatively unaffected in terms of their subjective wellbeing. These findings relate to the short-term, however, and it is possible that there may be delayed impacts on mental health over time. Trajectories over a longer time period will be further explored in the near future using Born in Bradford data for subsequent waves of these surveys. In addition, this study cautiously highlights the potential impact of social stressors present pre-pandemic and those children who may be more at risk of a deterioration in wellbeing now that schools are open again. It suggests that children of Pakistani heritage, as well as those children who felt excluded by others before the pandemic, may have experienced improved wellbeing at home compared to in school, which may also relate to the presence of protective factors not discovered here.

Further investigation on the basis of these findings is warranted to understand how best to ensure that subjective wellbeing does not decline to pre-pandemic levels for these children and also to understand any positive factors that may have led to improved wellbeing at home. It is not clear from the study why the changes observed differed between children of Pakistani heritage and White British backgrounds but it is possible some of these differences may be due to cultural and other differences in family environments, for example, household composition, as well as experiences of the school environment and other factors. Similarly, were the UK to enter a further lockdown to curb the spread of COVID-19 or other highly transmissible viruses in the future, these findings suggest that male children may be more at risk of adverse effects on their mental wellbeing and further evidence is needed to determine the factors associated with these effects.

Evidence from the YoungMinds survey undertaken at various timepoints during the pandemic, suggests that children with existing mental health needs may be more at risk of deteriorating mental health during the pandemic ( YoungMinds, 2021) and cross-sectional survey data from adolescents suggests females, those who had experienced food poverty and who had previously accessed mental health support were at greatest risk of depression, anxiety and deteriorating wellbeing ( Mansfield *et al.*, 2021). We could not explore the role of pre-pandemic baseline mental health problems on the wellbeing of children in our sample, but this perhaps indicates an area for future research.

For the children in this study, those who felt left out at school reported improvements to their wellbeing at home, suggesting that some of the harmful effects of negative in-person social interactions were less likely to impact during home learning and in the context of the rise in the use of online forms of communication during the pandemic. It is possible that these findings are explained by the comparatively young age range of the children in this sample who may be less likely than older children to use social media. In addition, the age range of children in this sample may be one of the reasons that wellbeing remained relatively unaffected overall. A recent, large systematic review exploring impacts on child and adolescent mental health during COVID-19, and covering 116 studies with data from 127,923 children and young people, found that older adolescents were the most likely to report negative changes to their mental health during the pandemic ( Samji *et al.*, 2021). Similarly, among adolescents (11–16), Cooper *et al.* (2021) found that during the pandemic, those with closer relationships with their parents reported less severe symptoms of mental health difficulties and lower levels of loneliness.

Policy and practice responses post-pandemic should recognise that the impact of the pandemic has caused a worsening of child wellbeing for some children. Interventions to identify and support these children are important to prevent longer-term and potentially more severe mental health difficulties from emerging. Of equal concern is the fact that some children had low wellbeing before the pandemic that improved when sheltered from the challenges of the school environment. Intervention is also needed for these children which should focus on ensuring that all children feel safe and included in the school environment and on identifying the specific protective factors that have proven positive for some children at home during the pandemic. 

### Strengths and limitations

The unique longitudinal cohort data available through the Born in Bradford research programme means that this study has been able to generate data for approximately 500 children from a diverse range of ethnic and socioeconomic backgrounds, and to compare child wellbeing outcomes over time during COVID-19, using a pre-pandemic baseline. The pandemic and the after-effects are likely to continue to affect the UK population for some time, and this research has generated evidence on the children most likely to be affected, as well as contributing to the evidence base on the role of demographic factors, social relationships and physical activity on child wellbeing more broadly.

A limitation of this study is that the PSY survey and the Phase 1 COVID Survey were administered by different methods, PSY was undertaken in educational settings, whilst the Phase 1 COVID Survey was posted out to children to complete at home. It is possible this may have affected responses, for example, children may be more reticent to report negative emotions if they are aware their parent or carer may read their survey before it is returned, or vice versa when completing the pre-pandemic survey close to their peers. This was unavoidable given the unique and challenging circumstances in which the survey was designed and carried out.

In addition, statistical analysis of these data was made more challenging by the comparatively small number of children who reported any changes to their feelings of happiness and (particularly) sadness between the two time points. This is, in itself, interesting and further supports the findings that most children experienced little change to their wellbeing during the pandemic. The low participation rates compared to the BiB PSY survey are potentially problematic as we found, for example, differences in ethnicity and IMD between the two samples, meaning that the findings reported here might not be representative of all children in the larger BiB sample. Nevertheless, these findings report wellbeing in a relatively large number of ethnically diverse children and we have been able to compare pre-pandemic to pandemic responses which is a strength of this study. Further research, using a larger sample of children would enable a more robust data analysis. This is likely to become more feasible as data collected during different phases of the pandemic becomes increasingly available, nationally and via additional surveys administered as part of Born in Bradford. Qualitative data collected as part of the BiB Covid-19 response may also provide further detail on the underlying reasons for any differences in wellbeing. 

## Conclusion

Through analysis of cohort data derived from a socioeconomically diverse, multi-ethnic sample of children, this study has demonstrated that many children experienced little change to their wellbeing during the pandemic and that some became happier compared to beforehand. More evidence is now needed to understand why some children are impacted more than others, including the role played by social relationships and protective factors, and to ensure preventative action can be taken to promote positive child wellbeing in the future.

## Data availability

### Underlying data

Scientists are encouraged to make use of the BiB data, which are available through a system of managed open access.

Before you contact us, please make sure you have read our Guidance for Collaborators. Our BiB executive review proposals on a monthly basis and we will endeavor to respond to your request as soon as possible. You can find out about the different datasets in our Data Dictionary. If you are unsure if we have the data that you need please contact a member of the BiB team ( borninbradford@bthft.nhs.uk).

Once you have formulated your request please complete the ‘Expression of Interest’ form available here and send to borninbradford@bthft.nhs.uk

If your request is approved we will ask you to sign a Data Sharing Contract and a Data Sharing Agreement, and if your request involves biological samples we will ask you to complete a material transfer agreement.

### Extended data

Harvard Dataverse: Changes in children’s wellbeing in Bradford during COVID-19: The Born in Bradford COVID-19 longitudinal research study. https://doi.org/10.7910/DVN/M8AEBT ( Pybus, 2022).

This project contains the following extended data

- Supplementary data tables.doc (including the following data)

- Overall change in happiness and sadness between PSY and Covid-1 surveys
- Happy more often compared to no change (base) in wellbeing over time (RRR)
- Happy less often compared to no change (base) in wellbeing over time (RRR)
- Sad less often compared to no change (base) in wellbeing over time (RRR)
- Sad more often compared to no change (base) in wellbeing over time (RRR)

- Me and my life survey.doc (survey questionnaire for PSY study)
- MID_BIB002_COVID_19_Child_Questionnaire_Phase 1.pdf (survey questionnaire for COVID-19 phrase 1 survey)

Data are available under the terms of the Creative Commons Zero "No rights reserved" data waiver (CC0 1.0 Public domain dedication).
