# Supplementary material for: Changes in children’s wellbeing in Bradford during COVID-19: The Born in Bradford COVID-19 longitudinal research study
Source: Wellcome Open Res. 2022 May 30;7:64. Originally published 2022 Feb 17. [Version 2] doi: 10.12688/wellcomeopenres.17642.2 (PMC9971641; doi:10.12688/wellcomeopenres.17642.2)
Supplement: Peer review history

# Peer review: Reviewer response for version 2

**DOI:** 10.21956/wellcomeopenres.19839.r50830

**Published:** 2023-02-01

**Authors:** Susan Woolfenden

**Affiliations:** 1 Population Child Health, School of Women's and Children's Health, Faculty of Medicine, University of New South Wales, Sydney, NSW, Australia

I am happy with the revisions made and no further changes are required from me.

Is the work clearly and accurately presented and does it cite the current literature?

Yes

If applicable, is the statistical analysis and its interpretation appropriate?

Yes

Are all the source data underlying the results available to ensure full reproducibility?

Yes

Is the study design appropriate and is the work technically sound?

Yes

Are the conclusions drawn adequately supported by the results?

Yes

Are sufficient details of methods and analysis provided to allow replication by others?

Yes

Reviewer Expertise:

NA

I confirm that I have read this submission and believe that I have an appropriate level of expertise to confirm that it is of an acceptable scientific standard.

# Peer review: Reviewer response for version 1

**DOI:** 10.21956/wellcomeopenres.19518.r49432

**Published:** 2022-03-31

**Authors:** Nicholas Spencer

**Affiliations:** 1 Warwick Medical School, University of Warwick, Coventry, UK

This paper is a valuable contribution to the literature on the impact of the pandemic on children’s wellbeing. It uses two Born in Bradford (BiB) studies, the on-going BiB Growing Up (BiBGU) and the BiB Primary School Years (BiBPSY), to compare children’s wellbeing before and during the early phase of the pandemic. Birth cohort studies tend to enroll and retain children from more affluent and majority ethnic families; however, the cohort recruited and retained by BiB has a strong representation of families in the more deprived areas of the city of Bradford and those of Pakistani heritage.   

 The findings of this study suggest that, contrary to many predictions, children reported being happier and less sad during the early phase of the pandemic compared with the pre-pandemic period. The finding that children of Pakistani heritage were likely to feel sad less often than those of white British heritage is striking and worthy of more detailed examination particularly as the prevalence rate of Covid cases and deaths among adults has been high in this minority ethnic group compared with the white British majority.  Living in deprived areas in the UK has been associated with high Covid prevalence and death rates compared with more affluent areas but this study found no difference in children’s wellbeing in the pandemic by area deprivation.   

 Although the study methodology is generally robust, I think the paper could be further strengthened if the following issues are addressed:

1. The low participation rate is a threat to the validity of the study and, although the authors compare the characteristics of the study sample with the BiBPSY sample, I think the limitations associated with the low rate should be considered in the relevant section of the discussion.
2. The data on the main outcomes of interest (changes in happiness and sadness) are based on questions derived from the Millennium Cohort Study, the International Survey of Children’s Wellbeing and the Avon Longitudinal Study of Parents and Children. The paper by Pickett et al cited as reference mentions a test of understanding and face validity but I think specific information on whether these questions were tested for validity in the three source surveys, especially in view of their use in an ethnically diverse population, should be included in the methods.
3. The Covid 19 questionnaires were completed in May-July 2020 during the first national UK lockdown. For international readers, it would be useful to specify the period of time the children studied had been excluded from school and other activities involving social interaction prior to completing the survey.
4. A minor clarification – the age range of the children surveyed is given as 7-13 in the opening paragraph of the methods but ages are limited to 7-10 in Figures 1 & 2 and in table 4. This should be corrected or clarified.   
5. I agree with the conclusion that more evidence is needed to fully understand these findings but I think the authors should indicate how this might be obtained.

Is the work clearly and accurately presented and does it cite the current literature?

Yes

If applicable, is the statistical analysis and its interpretation appropriate?

Yes

Are all the source data underlying the results available to ensure full reproducibility?

Yes

Is the study design appropriate and is the work technically sound?

Partly

Are the conclusions drawn adequately supported by the results?

Yes

Are sufficient details of methods and analysis provided to allow replication by others?

Yes

Reviewer Expertise:

Child public health and social determinants of child health

I confirm that I have read this submission and believe that I have an appropriate level of expertise to confirm that it is of an acceptable scientific standard.

# Response

**Published:** 2022-05-30

**Authors:** Katie Pybus

**Affiliations:** University of York, UK, UK

This paper is a valuable contribution to the literature on the impact of the pandemic on children’s wellbeing. It uses two Born in Bradford (BiB) studies, the ongoing BiB Growing Up (BiBGU) and the BiB Primary School Years (BiBPSY) to compare children’s wellbeing before and during the early phase of the pandemic. Birth cohort studies tend to enrol and retain children from more affluent and majority ethnic families; however, the cohort recruited and retained by BiB has a strong representation of families in the more deprived areas of the city of Bradford and those of Pakistani heritage.

**Response: Thank you for your helpful review. We have made changes to the manuscript in line with the suggestions and hope that this has strengthened the article.**

 The findings of this study suggest that, contrary to many predictions, children reported being happier and less sad during the early phase of the pandemic compared with the pre-pandemic period. The finding that children of Pakistani heritage were likely to feel sad less often than those of white British heritage is striking and worthy of more detailed examination particularly as the prevalence rate of Covid cases and deaths among adults has been high in this minority ethnic group compared with the White British majority. Living in deprived areas in the UK has been associated with high Covid prevalence and death rates compared with more affluent areas but this study found no difference in children’s wellbeing in the pandemic by area deprivation. Although the study methodology is generally robust, I think the paper could be further strengthened if the following issues are addressed:

 The low participation rate is a threat to the validity of the study and, although the authors compare the characteristics of the study sample with the BiBPSY sample, I think the limitations associated with the low rate should be considered in the relevant section of the discussion.

**Response: We have added additional discussion on the low participation rate to the ‘Strengths and Limitations section’ of the discussion. Please see paragraph 3 of this section.**

 The data on the main outcomes of interest (changes in happiness and sadness) are based on questions derived from the Millennium Cohort Study, the International Survey of Children’s Wellbeing and the Avon Longitudinal Study of Parents and Children. The paper by Pickett et al cited as reference mentions a test of understanding and face validity but I think specific information on whether these questions were tested for validity in the three source surveys, especially in view of their use in an ethnically diverse population, should be included in the methods.

**Response: We have included additional text to further explain the piloting process undertaken during development of the Millennium Cohort Study and Primary School Years survey materials. Please see paragraph 1 of the ‘Study Measures’ section.**

 The Covid-19 questionnaires were completed in May-July 2020 during the first national UK lockdown. For international readers, it would be useful to specify the period of time the children studied had been excluded from school and other activities involving social interaction prior to completing the survey.

**Response: Clarification has been added to the ‘Setting’ section which we hope addresses this point. Please see paragraph 3 of this section.**

 A minor clarification – the age range of the children surveyed is given as 7-13 in the opening paragraph of the methods section but ages are limited to 7-10 in Figures 1&2 and in Table 4. This should be corrected or clarified.

**Response: Thank you for making us aware of this, we have clarified in the section titled ‘Multinomial logistic regression models’, please see paragraph 1 of this section.**

 I agree with the conclusion that more evidence is needed to fully understand these findings but I think the authors should indicate how this might be obtained.

**Response: We have added additional discussion on obtaining further evidence to the final paragraph of the 'Strengths and Limitations' section of the discussion. Please see paragraph 3 of this section.**

# Peer review: Reviewer response for version 1

**DOI:** 10.21956/wellcomeopenres.19518.r48810

**Published:** 2022-03-21

**Authors:** Susan Woolfenden

**Affiliations:** 1 Population Child Health, School of Women's and Children's Health, Faculty of Medicine, University of New South Wales, Sydney, NSW, Australia

Thank you for this very useful and interesting paper.

 I have only a few suggestions that I think would add to it: 

**Abstract:**

- Results - split each finding into its own sentence to avoid confusion

**Intro:**

- I think it would be worthwhile to define what you mean by wellbeing vs mental health - are they the same or different - are you choosing a more strengths based position with wellbeing - which is great but just clarify.
- While you talk about the gaps in the literature re the ethnicity data for children in the pandemic it is not made clear why this is important (i.e. a group more likely to experience inequity) from a research, policy and service provision standpoint.
- Also need to have in your rationale why you are looking at the quality of social relationships and what do you mean by that term.

**Methods:**

- I don't think it is made clear what you mean by quality of social relationships and what variables they are being measured by - it is implied but not stated

**Results:**

- Table 1 - you look like you have some stats significant differences in your responders vs the whole cohort so if you an I would explore that with P values.
- Can you also make it clearer when the prepandemic measures were measured in the methods? 

**Discussion:**

-  think the results are really interesting and more can be made of them in the discussion
- Why Pakistani children are doing well is because of strengths from that culture - collective, supportive, etc. - this could be explored.
- I think the implications of findings are clear for the research implications but this is also an opportunity for service and policy recommendations for beneficial childhood experiences as well - i.e. how do we build things better postpandemic?

Is the work clearly and accurately presented and does it cite the current literature?

Yes

If applicable, is the statistical analysis and its interpretation appropriate?

Yes

Are all the source data underlying the results available to ensure full reproducibility?

Yes

Is the study design appropriate and is the work technically sound?

Yes

Are the conclusions drawn adequately supported by the results?

Yes

Are sufficient details of methods and analysis provided to allow replication by others?

Yes

Reviewer Expertise:

Community Child Health

I confirm that I have read this submission and believe that I have an appropriate level of expertise to confirm that it is of an acceptable scientific standard, however I have significant reservations, as outlined above.

# Response

**Published:** 2022-05-30

**Authors:** Katie Pybus

**Affiliations:** University of York, UK, UK

Thank you for this very useful and interesting paper. I have only a few suggestions that I think would add to it.

**Response: Thank you for your helpful review. We have made changes to the manuscript in line with the suggestions and hope that this has strengthened the article.**

Abstract

 Results – split each finding into its own sentence to avoid confusion.

**Response: We have amended the results section of the abstract as suggested.**

Background

 I think it would be worthwhile to define what you mean by wellbeing vs mental health – are they the same or different – are you choosing a more strengths based position with wellbeing – which is great but just clarify.

**Response: We have added text to the introduction to clarify this point. Please see Background, paragraph 7.**

 While you talk about the gaps in the literature re the ethnicity data for children in the pandemic it is not made clear why this is important (i.e., a group more likely to experience inequity) from a research, policy and service provision standpoint.

**Response: We have added text to the introduction to clarify this point. Please see Background, paragraph 6.**

 Also need to have in your rationale why you are looking at the quality of social relationships and what do you mean by that term.

**Response: We have added text to the introduction to clarify this point. Please see Background, paragraph 7.**

Methods

 I don’t think it is made clear what you mean by quality of social relationships and what variables they are being measured by – it is implied but not stated.

**Response: We have added clarification to the ‘Study measures’ section of the methods, please see paragraph 6 of this section.**

Results

 Table 1 – you look like you have some stats significant differences in your responders vs the whole cohort, I would explore that with P values.

**Response: We welcome the suggestion to explore statistical significance of the differences reported in Table 1. We have calculated p-values associated with the differences, using chi squared tests for proportions, and t-test for differences in mean age. The associated p-values have been reported in a footnote to Table 1. We have added further discussion to the 'Strengths and limitations' section.**

 Can you also make it clearer when the pre-pandemic measures were measured in the methods?

**Response: We have included clarification of the pre-pandemic measurement dates in the ‘Study measures’ section of the methods. Please see paragraph 3 of this section.**

Discussion

 Think the results are really interesting and more can be made of them in the discussion. Why Pakistani children are doing well is because of strengths from that culture – collective, supportive, etc. – this could be explored.

**Response: Thank you for your suggestion, we have incorporated further discussion on this point to the ‘Implications of findings’ section. Please see paragraph 2 of this section.**

 I think the implications of findings are clear for the research implications but this is also an opportunity for service and policy recommendations for beneficial childhood experiences as well – i.e., how do we build things back better post-pandemic?

**Response: Thank you for your suggestion, we have added further discussion on these points to the ‘Implications of findings’ section. Please see paragraph 5 of this section.**
